# FOXK2 Transcription Factor Suppresses ERα-positive Breast Cancer Cell Growth Through Down-Regulating the Stability of ERα via mechanism involving BRCA1/BARD1

**DOI:** 10.1038/srep08796

**Published:** 2015-03-05

**Authors:** Ying Liu, Xiang Ao, Zhaojun Jia, Xiao-Yan Bai, Zhaowei Xu, Gaolei Hu, Xiao Jiang, Min Chen, Huijian Wu

**Affiliations:** 1 School of Life Science and Biotechnology, Dalian University of Technology, Dalian 116024, Liaoning, China; 2 School of Life Science and Medicine, Dalian University of Technology, Panjin 114221, Liaoning, China

## Abstract

Estrogen receptors (ERs) are critical regulators of breast cancer development. Identification of molecules that regulate the function of ERs may facilitate the development of more effective breast cancer treatment strategies. In this study, we showed that the forkhead transcription factor FOXK2 interacted with ERα, and inhibited ERα-regulated transcriptional activities by enhancing the ubiquitin-mediated degradation of ERα. This process involved the interaction between FOXK2 and BRCA1/BARD1, the E3 ubiquitin ligase of ERα. FOXK2 interacted with BARD1 and acted as a scaffold protein for BRCA1/BARD1 and ERα, leading to enhanced degradation of ERα, which eventually accounted for its decreased transcriptional activity. Consistent with these observations, overexpression of FOXK2 inhibited the transcriptional activity of ERα, decreased the transcription of ERα target genes, and suppressed the proliferation of ERα-positive breast cancer cells. In contract, knockdown of FOXK2 in MCF-7 cells promoted cell proliferation. However, when ERα was also knocked down, knockdown of FOXK2 had no effect on cell proliferation. These findings suggested that FOXK2 might act as a negative regulator of ERα, and its association with both ERα and BRCA1/BARD1 could lead to the down-regulation of ERα transcriptional activity, effectively regulating the function of ERα.

Breast cancer, the most common form of malignant disease among women, has become the second leading cause of cancer death^1^. The common risk factors for breast cancer include family history, reproductive factors, dietary factors and estrogen^2^. Among them, estrogen has been recognized as a key carcinogenic factor in the initiation and progression of breast cancer. Longer exposures to estrogen result in an increased risk of breast cancer^3^. Estrogen exerts its physiological function through binding with ERs, which then forms a dimer and binds to estrogen-responsive elements (EREs) in the promoters of the target genes to regulate their expressions. There are two isoforms of ERs, and these are ERα and ERβ. ERα is closely associated with the development of ER-positive breast cancer^4^. Nearly 70% of breast cancer express ERα and are estrogen dependent^5^. Clinically, ERα is viewed as a valuable predictive and prognostic factor for breast cancer treatment. Consequently, inhibition of ERα has become one of the major strategies for the prevention and treatment of breast cancer. Currently, ERα is a major target for endocrine therapy^6^. Multiple cellular and molecular events can regulate ERα function, such as genic mutation, epigenetic modification, or direct interaction with corepressor proteins that repress ER-α-mediated transcriptional activity^7^. However, the detailed mechanism involved in the regulation of ERα function is still inconclusive, and this appears to restrict our understanding on the pathogenesis of ERα-positive breast cancer. Thus it is extremely important to gain further insight into how ERα function is regulated.

Numerous studies have shown that ERα is tightly regulated by post-translational modifications (PTMs), such as phosphorylation, methylation, acetylation, sumoylation and ubiquitination^8^,^9^,^10^,^11^,^12^. In these PTMs, ubiquitination can down-regulate the protein level of ERα and suppress its transcriptional activity^13^. Ubiquitination involves several steps and three well-known enzymes called ubiquitin activating enzyme (E1), ubiquitin conjugating enzyme (E2), and ubiquitin ligases (E3). Among the three enzymes, only E3 ubiquitin ligases physically interact with their substrates, and therefore confer some degree of specificity. Several E3 ubiquitin ligases are known to associate with the ubiquitination of ERα, include the C terminus of Hsc70-interacting protein (CHIP), Breast cancer type 1 susceptibility protein (BRCA1)/BRCA1-associated RING domain protein 1 (BARD1), murine double minute 2 (MDM2) and ring finger protein (RNF31)^13^,^14^,^15^,^16^. Among them, BRCA1/BARD1 complex is a well-known E3 ubiquitin ligase, and it has been widely investigated. BRCA1/BARD1 plays important roles in DNA-damage response and tumor suppression through degrading a set of substrates such as RNA pol II and FANCD2 in addition to ERα^17^.

Forkhead box K2 (FOXK2), also known as ILF or ILF1, is one of the forkhead transcription factors that contain a conserved forkhead winged helix-turn-helix DNA binding domain (FOX domain). It was first identified as a regulator of IL-2 transcription, where it acts as a transcriptional repressor^18^. In common with other forkhead transcription factors, FOXK2 contains a FOX domain in addition to a FHA domain that mediates its interaction with other proteins. The function of FOXK2 is regulated by the CDK1/Cyclin B kinase complex which modulates its stability and activity^19^. FOXK2 interacts with AP-1, and promotes the binding of AP-1 to chromatin, resulting in the up-regulation of AP-1-dependent gene expression^20^. It can also bind to G/T-mismatch DNA and initiate the process of DNA mismatch repair^21^. FOXA1, another member of the forkhead transcription factors, has been shown to interact with ERα via its FOX domain, and mediates the recruitment of ERα to chromatin, leading to the up-regulation of ERα target genes^22^. Other members of this protein family, such as FOXO3a can also interact with ERα via its FOX domain, but its action inhibits ERα transcriptional activities, causing a down-regulation in the expression of ERs target genes, and suppression of the proliferation of ERα-positive breast cancer cells^6^. Both FOXA1 and FOXO3a can interact with ERα via their FOX domains, but they exert completely different effects on the regulation of ERα. This suggests that FOX domain just mediates the interaction of forkhead proteins with ERα, whereas other domains in their structures may affect ERα function. FOXK2 contains a conserved FOX domain, and our preliminary data have shown that FOXK2 can inhibit the transcriptional activity of ERα. We therefore wanted to know whether FOXK2 can interact with ERα and regulate its function.

In this report, we observed a negative correlation between ERα and FOXK2 in human breast cancer. We demonstrated that FOXK2 could interact with ERα via the region containing FOX domain (amino acids 128 to 353), leading to lower protein stability for ERα, and inhibition of its transcriptional activity. Such regulation of ERα by FOXK2 occurred via a mechanism that involved BRCA1/BARD1. We also showed that FOXK2 could suppress ERα-mediated proliferation of breast cancer cells. Taken together, our data suggested that FOXK2 might act as a negative regulator of ERα.

## Results

### FOXK2 is associated with ERα in human breast cancer

**Figure 1:**
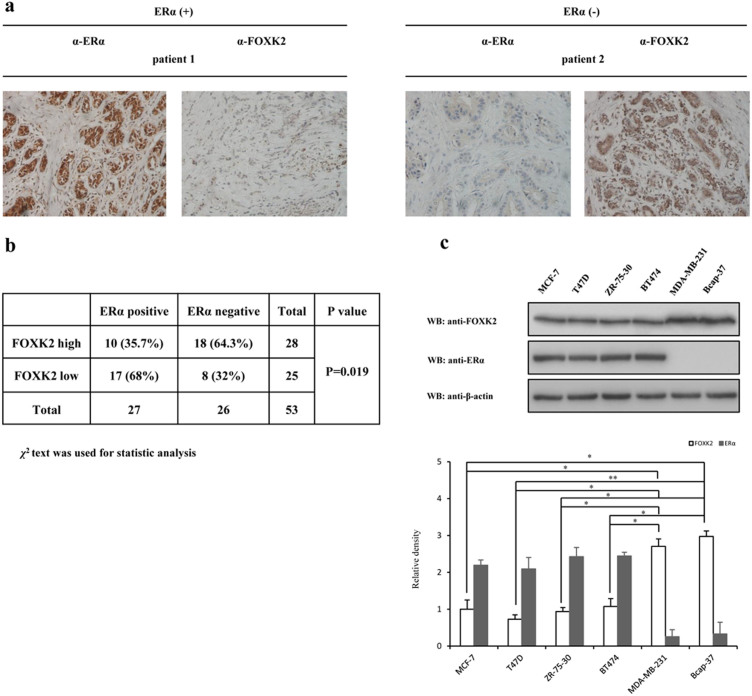
Correlation between ERα and FOXK2 in human breast cancer. (a) Representative results showing the immunohistochemical staining of ERα and FOXK2 in sections of the breast tumor tissues. Each sample was incubated with antibody against ERα or FOXK2. Positive staining and negative staining are indicated by brown and blue staining, respectively (×200 Magnification). (b) Correlation between ERα and FOXK2 expression suggested by the 53 breast cancer specimens. χ^2^ test was used for statistical analysis. P values less than 0.05 were considered to indicate statistical significance. (c) Western blot analysis comparing the endogenous FOXK2 protein levels in several breast cancer cells. Experiments were repeated at least three times. The level of FOXK2 protein from MCF-7 cells was set to 1. Data shown in the graphs are the means ± SDs of three experiments. *, P < 0.05; **, P < 0.01. The full-length blot of Figure 1 is presented in Supplementary Figure 1.

Aberrant ERα signaling is known to play an important role in the occurrence of ERα-positive breast cancer. However, little is known about the role of FOXK2 in tumorigenesis of breast cancer. In order to examine the relationship between FOXK2 and ERα in breast cancer, we compared the protein levels of FOXK2 and ERα in the breast cancer specimens (Fig. 1a). A total of 53 breast tumor specimens (27 ERα-positive and 26 ERα-negative) were analyzed by immunohistochemical assay. According to the comparison of H-score, seventeen of the ERα-positive samples (63%) showed low FOXK2 expression, whereas only eight of the ERα-negative samples (31%) showed low FOXK2 expression (Fig. 1b). We also examined the protein levels of endogenous ERα and FOXK2 in various breast cancer cell lines. As shown in Fig. 1c, the levels of FOXK2 expression in the ERα-positive breast cancer cell lines (MCF-7, T47D, ZR-51-30 and BT474) were significantly lower than that in ERα-negative breast cancer cell lines (MDA-MB-231 and Bcap-37). Taken together, these results suggested that a negative correlation existed between ERα and FOXK2 in breast cancer.

### FOXK2 interacts with ERα in breast cancer cells

**Figure 2:**
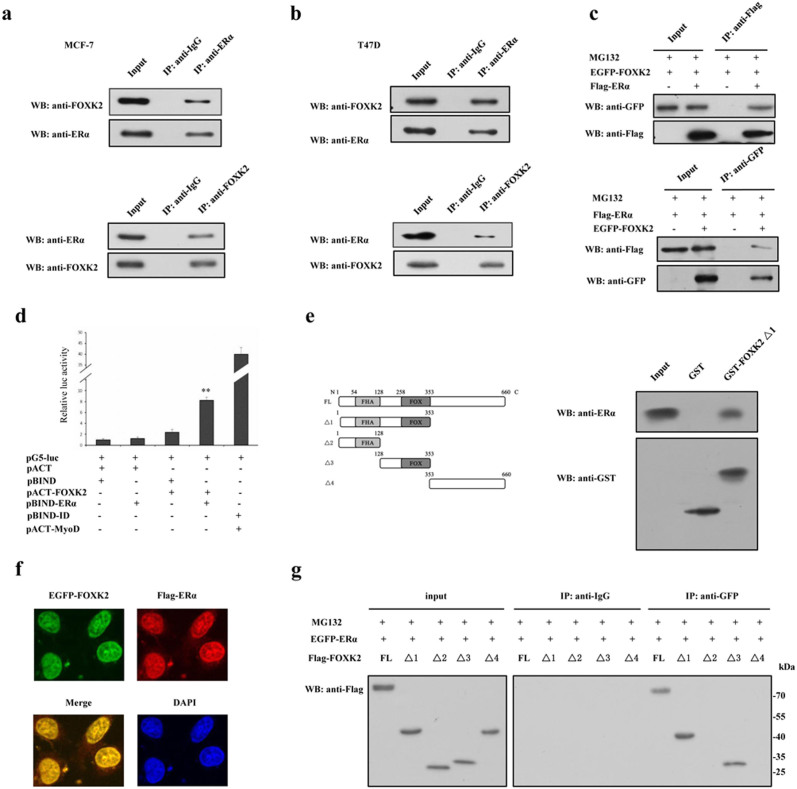
Interaction between FOXK2 and ERα. (a–b) MCF-7 and T47D cells were subjected to immunoprecipitation with anti-ERα antibody followed by Western blot with anti-FOXK2 antibody or vice versa. Immunoprecipitation carried out with anti-IgG antibody was used as control. (c) HEK 293T cells transfected with EGFP-tagged FOXK2 only, or with EGFP-tagged FOXK2 and Flag-tagged ERα were subjected to immunoprecipitation with anti-Flag antibody followed by Western blot with anti-GFP antibody or vice versa. (d) The interaction between FOXK2 and ERα was detected using a mammalian two hybrid system. FOXK2 and ERα were expressed from pBIND-ERα and pACT-FOXK2, respectively, whereas the empty vectors pACT and pBIND were expressed as controls, as indicated with the pG5-luc reporter in HEK 293T cells. Cells were transfected with pBIND-ID and pACT-MyoD as a positive control. Luciferase activity was measured 36 h after transfection. The luc activity level of cells transfected with pG5-luc, pACT and pBIND was set to 1. Data shown in the graphs are the means ± S.Ds of three experiments. **, P < 0.01 compared with cells transfected with pACT and pBIND. (e) Interaction of FOXK2 with ERα *in vitro*. Extract of MCF-7cells were incubated with immobilized GST-FOXK2 Δ1 or GST alone. The bound proteins were subjected to Western blot assay. (f) Localization of ERα and FOXK2 in MCF-7. MCF-7 cells transfected with EGFP-FOXK2 and Flag-ERα were stained with rabbit anti-Flag antibody and tetraethyl rhodamine isothiocyanate (TRITC)-conjugated anti-rabbit IgG. EGFP-FOXK2 appeared as green signal when visualized by fluorescence microscopy. Nuclei were stained with 4,6-diamidino-2-phenylindole (DAPI). (g) HEK 293T cells were transfected with EGFP-tagged ERα and Flag-tagged full-length FOXK2 (FL), FOXK2 Δ1, FOXK2 Δ2, FOXK2 Δ3 or FOXK2 Δ4 and then treated with 10 μM MG132 for 8 h. The cells were subjected to immunoprecipitation with anti-IgG or anti-GFP antibody followed by Western blot with anti-Flag antibody. All experiments were repeated at least three times. The full-length blot of Figure 2 is presented in Supplementary Figure 2.

In breast cancer cells, FOXA1 and FOXO3a can regulate the function of ERα via their FOX domains, and since FOXK2 also contains a conserved FOX domain, we speculated that it too may interact with ERα. In order to investigate this possibility, we performed co-immunoprecipitation experiments in two different cell lines (MCF-7 and T47D) using either anti-FOXK2 or anti-ERα antibody. A positive interaction between endogenous FOXK2 and ERα was evident in MCF-7 and T47D cells (Figs. 2a and 2b). Similar co-precipitation of FOXK2 and ERα were obtained when the same immunoprecipitation experiment was carried out in HEK 293T cells that were transfected with EGFP-FOXK2 and Flag-ERα (Fig. 2c). The interaction between FOXK2 and ERα was confirmed using mammalian two-hybrid system. Transactivation by pBIND–ERα was evident by co-expressing a pACT–FOXK2 fusion protein (Fig. 2d). Moreover, GST pull-down assay further confirmed the interaction between FOXK2 Δ1 and ERα in vitro (Fig. 2e). Double-label fluorescence immunohistochemistry carried out in MCF-7 cells showed that both of FOXK2 and ERα were localized in the nucleus (Fig. 2f), further strengthening our speculation that FOXK2 may directly participate in the estrogen signaling pathway. To elucidate the region of FOXK2 that might mediate the interaction between FOXK2 and ERα, HEK 293T cells were transfected with EGFP-tagged ERα together with Flag-tagged full-length FOXK2 (FOXK2 FL) or mutant FOXK2 (FOXK2 Δ1 contained FHA and FOX domains, FOXK2 Δ2 contained FHA domain, FOXK2 Δ3 contained FOX domain and FOXK2 Δ4 contained C-terminal tail domain). The transfected cells were subjected to immunoprecipitation carried out with anti-GFP antibody, followed by Western blot with anti-Flag antibody. Positive interactions were obtained only between ERα and FOXK2 FL or Δ1 or Δ3, but not with ERα and FOXK2 Δ2 or Δ4 (Fig. 2g), indicating that the region containing the FOX domain (amino acids 128 to 353) mediated the interaction between FOXK2 and ERα, and probably exerted an important effect on the regulation of ERα.

### FOXK2 decreases ERα protein level by promoting its ubiquitin-dependent degradation

**Figure 3:**
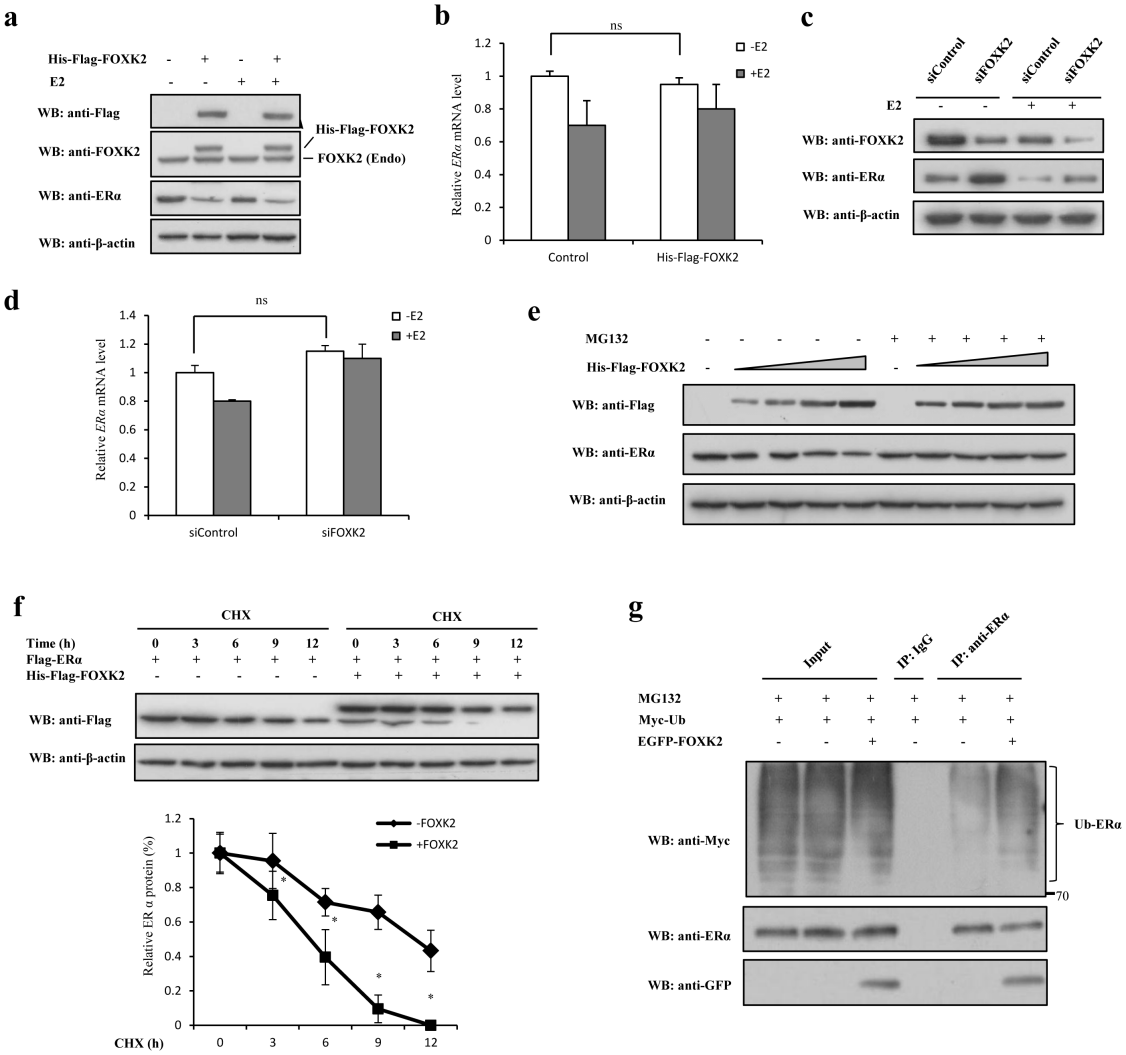
Effect of FOXK2 on ubiquitin-dependent degradation of ERα. (a) HEK 293T cells transfected with Flag-ERα only or with Flag-ERα and His-Flag-FOXK2 were treated with or without 10 nM E2 for 16 h. The samples were subjected to Western blot analysis with the indicated antibodies. (b) MCF-7 cells transfected with empty vector or His-Flag-FOXK2 were treated with or without 10 nM E2 for 16 h. The samples were subjected to Realtime-PCR analysis. For comparison, the level of ERα mRNA for the control MCF-7 cells was set to 1. (c) MCF-7 cells transfected with siFOXK2 pool or siControl were treated with or without 10 nM E2 for 16 h. The samples were subjected to Western blot analysis with the indicated antibodies. (d) MCF-7 cells transfected with siFOXK2 pool or siControl were treated with or without 10 nM E2 for 16 h. The samples were subjected to Realtime-PCR analysis. For comparison, the level of ERα mRNA for the control MCF-7 cells was set to 1. (e) HEK 293T cells transfected with different doses of His-Flag-FOXK2 (0, 1, 2, 4 and 6 μg) were treated with or without 10 μM MG132 for 8 h. The samples were subjected to Western blot analysis with the indicated antibodies. (f) HEK 293T cells transfected with Flag-ERα only, or Flag-ERα and His-Flag-FOXK2 were treated with 10 μg/ml cycloheximide (CHX) for different periods of time (0, 3, 6, 9, 12 h) before being subjected to Western blot. The graph shows the relative intensity of the ERα band at different time points. The level of ERα protein for control HEK 293T cells was set to 1. (g) MCF-7 cells transfected with Myc-Ub only or with Myc-Ub and EGFP-FOXK2 were treated with 10 μM MG132 for 8 h. The samples were subjected to immunoprecipitation with anti-IgG or anti-ERα antibody followed by Western blot analysis with anti-Myc antibody. All experiments were repeated at least three times. Data shown in the graphs are the means ± SDs of three experiments. *, P < 0.05; ns, not significant. The full-length blot of Figure 3 is presented in Supplementary Figure 3.

Given that there was a negative correlation between ERα and FOXK2 in human breast cancer, we speculated that there may be a causal relationship between FOXK2 and ERα at the protein level. To investigate this possibility, MCF-7 cells were transfected with a control vector or His-Flag-FOXK2 and their endogenous levels of ERα were compared by Western blot. Overexpression of FOXK2 resulted in reduced ERα protein level (Fig. 3a). Considering that this could be also due to changes in level of ERα transcript, changes in ERα mRNA levels in MCF-7 cells were then examined. Real-time PCR analysis showed that overexpression of FOXK2 had no effect on the level of ERα mRNA (Fig. 3b). Furthermore, knockdown of FOXK2 by siRNA pool (with four individual siRNAs targeting *FOXK2* gene) increased the endogenous ERα protein level (Fig. 3c) without changing the level of ERα mRNA in MCF-7 cells (Fig. 3d), suggesting that the reduced level of ERα protein caused by FOXK2 was due to the change in ERα protein stability. Considering the stability of ERα is known to be regulated by proteasome-mediated degradation^23^,^24^, the effect of overexpression of FOXK2 on the stability of ERα was further examined in the absence and presence of MG132, a proteasome inhibitor. In the absence of MG132, the protein level of endogenous ERα decreased with increasing dosages of FOXK2, whereas in the presence of MG132, the levels were similar among regardless of the dosages of FOXK2 (Fig. 3e), suggesting that MG132 could inhibit the proteasome-dependent degradation of ERα promoted by FOXK2. The half-life of ERα in MCF-7 cells transfected with or without wild-type FOXK2 was determined after the cells were treated with cycloheximide, an inhibitor of protein biosynthesis. The results demonstrated that overexpression of FOXK2 shortened the half-life of ERα from 11 h to 5 h (Fig. 3f) and increased the ubiquitination of ERα (Fig. 3g). Taken together, these results suggested that FOXK2 could decrease the stability of ERα through promoting its ubiquitin-dependent degradation.

### FOXK2 interacts with BARD1 and increases the ubiquitination of ERα

**Figure 4:**
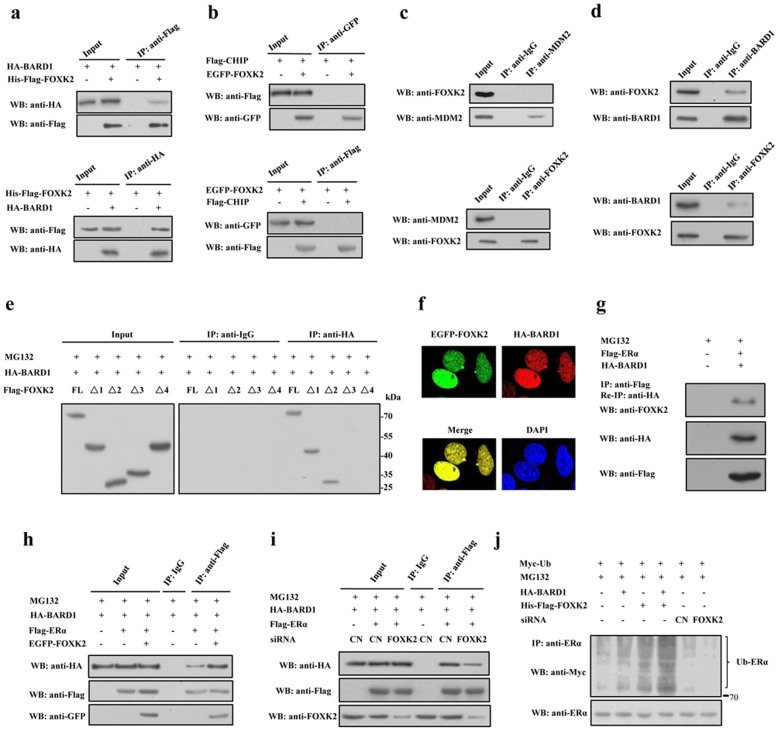
Effect of FOXK2 on the interaction between BARD1 and ERα. (a) HEK 293T cells transfected with HA-BARD1 only, or with HA-BARD1 and His-Flag-FOXK2 were subjected to immunoprecipitation with anti-Flag antibody followed by Western blot with anti-HA antibody or vice versa. (b) HEK 293T cells transfected with appropriate plasmids were subjected to immunoprecipitation and Western blot with specific antibodies as indicated or vice versa. (c) MCF-7 cells were subjected to immunoprecipitation with anti-MDM2 antibody followed by Western blot with anti-FOXK2 antibody or vice versa. (d) MCF-7 cells were subjected to immunoprecipitation with anti-BARD1 antibody followed by Western blot with anti-FOXK2 antibody or vice versa. (e) HEK 293T cells transfected with HA-BARD1 and different FOXK2 constructs as indicated were collected and then subjected to immunoprecipitation with anti-HA antibody, followed by Western blot analysis with anti-Flag antibody. (f) MCF-7 cells transfected with EGFP-FOXK2 and HA-BARD1 were stained with rabbit anti-HA antibody and TRITC-conjugated anti-rabbit IgG, and then counterstained with DAPI (blue) for nucleus detection. EGFP-FOXK2 appeared as green signal when visualized by fluorescence microscopy. (g) MCF-7 cells transfected with Flag-ERα and HA-BARD1 were subjected to immunoprecipitation with anti-Flag antibody and re-immunoprecipitation (RE) with anti-HA antibody followed by Western blot with anti-FOXK2 antibody, anti-Flag or anti-HA antibody. (h) HEK 293T cells transfected with appropriate plasmids as indicated, and then treated with 10 μM MG132 for 8 h. Cells were collected and then subjected to immunoprecipitation with anti-Flag antibody followed by Western blot with the indicated antibodies. (i) HEK 293T cells transfected with appropriate plasmids as indicated, and then treated with 10 μM MG132 for 8 h. Cells were collected and then subjected to immunoprecipitation with anti-Flag antibody followed by Western blot with the indicated antibodies. (j) HEK 293T cells transfected with various combinations of different constructs as indicated were treated with 10 μM MG132 for 8 h. The cells were collected and subjected to immunoprecipitation with anti-ERα antibody followed by Western blot analysis with anti-Myc antibody. All experiments were repeated at least three times. The full-length blot of Figure 4 is presented in Supplementary Figure 4.

Protein sequence analysis showed that FOXK2 did not have the RING, U-box and HECT domains, which are catalytic domains of ubiquitin E3 ligase, and therefore FOXK2 may not function as an ubiquitin E3 ligase. So we speculated that FOXK2 may increase the ubiquitination of ERα through interaction with other E3 ligases. In order to examine which ubiquitin E3 ligase is involved in FOXK2-promoted ubiquitination of ERα, we examined the interaction between FOXK2 and the ubiquitin E3 ligases of ERα. Co-immunoprecipitation experiments revealed a positive interaction between FOXK2 and BARD1 (Fig. 4a), whereas no interaction between FOXK2 and CHIP or FOXK2 and MDM2 was observed (Fig. 4b and 4c), suggesting that BRCA1/BARD1 might participate in FOXK2-mediated degradation of ERα. To further confirm the interaction of FOXK2 with BARD1, we performed co-immunoprecipitation experiment using MCF-7 cells and either anti-FOXK2 or anti-BARD1 antibody. A positive interaction between endogenous FOXK2 and BARD1 was observed (Fig. 4d). To map the region of FOXK2 that interacted with BARD1, we performed the same experiment for the different truncated FOXK2 mutants. Positive interaction was seen between BARD1 and FOXK2 FL or Δ1 or Δ2, whereas no interaction was detected between BARD1 and FOXK2 Δ3 or Δ4 (Fig. 4e), suggesting that the interaction of FOXK2 and BARD1 was mediated by the amino-terminal region containing FHA domain (amino acids 1 to 128) of FOXK2. Double-label fluorescence immunohistochemistry further revealed that both FOXK2 and BARD1 were localized in the nucleus of the cell (Fig. 4f). Given that FOXK2 could interact with both ERα and BARD1, we speculated that an ERα-FOXK2-BARD1 complex might exist. To investigate this possibility, we performed re-immunoprecipitation and Western blot assay. A band was detected when extract of MCF-7 cells that overexpressed Flag-ERα and HA-BARD1 was probed with anti-FOXK2 antibody (Fig. 4g), indicating the existence of ERα-FOXK2-BARD1 complex. We further examined whether FOXK2 could affect the interaction of ERα with BARD1. The results showed that overexpression of FOXK2 enhanced the interaction between ERα and BARD1 (Fig. 4h), whereas knockdown of FOXK2 by siRNA decreased this interaction (Fig. 4i), indicating that FOXK2 probably acted as a scaffold protein to enhance the interaction of BRCA1/BARD1 with ERα. Next, we examined the effect of FOXK2 on BARD1-mediated ubiquitination of ERα. As shown in Fig. 4j, both FOXK2 and BARD1 enhanced the ubiquitination of ERα, with the extent of ubiquitination being enhanced when both FOXK2 and BARD1 were overexpressed. In contrast, knockdown of FOXK2 decreased the ubiquitination of ERα. Taken together, these results suggested that FOXK2 probably facilitated the interaction of ERα with its ubiquitin E3 ligase BRCA1/BARD1 complex, therefore, promoting the ubiquitin-mediated degradation of ERα.

### FOXK2 suppresses the transcriptional activity of ERα

**Figure 5:**
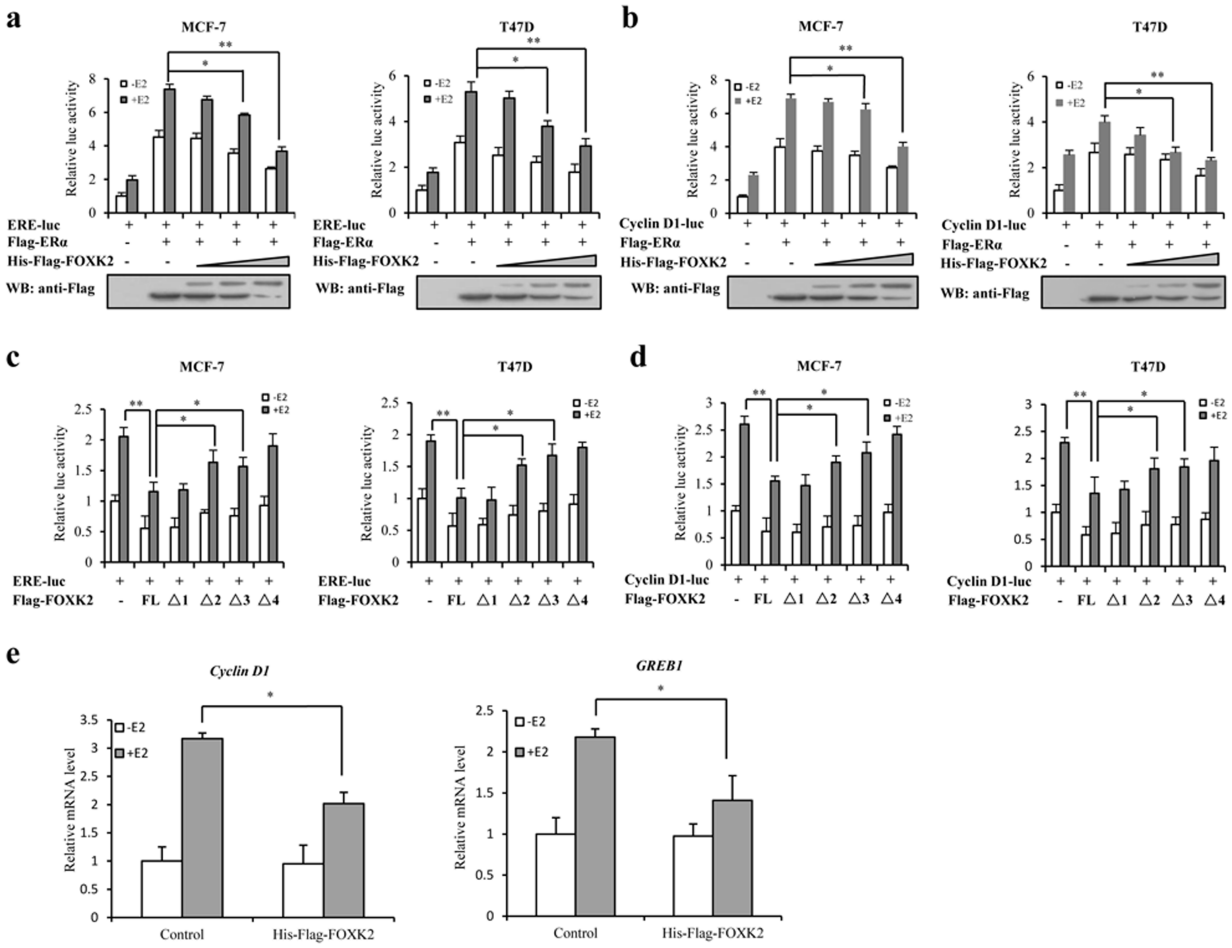
Effect of FOXK2 on the transcriptional activity of ERα and its downstream target genes. (a) MCF-7 and T47D cells were transfected with ERE-luciferase, Flag-ERα and His-Flag-FOXK2. Luciferase activity was measured either with or without pre-treatment of the cells with 10 nM E2 for 16 h. For comparison, the ERE-luc activity level of control cells was set to 1. (b) MCF-7 and T47D cells were transfected with Cyclin D1-luciferase with Flag-ERα and His-Flag-FOXK2. Luciferase activity was measured either with or without pre-treatment of the cells with 10 nM E2 for 16 h. For comparison, the Cyclin D1-luc activity level of control cells was set to 1. (c) MCF-7 and T47D cells were transfected with ERE-luciferase and different FOXK2 constructs. Luciferase activity was measured either with or without pre-treatment of the cells with 10 nM E2 for 16 h. For comparison, the ERE-luc activity level of control cells was set to 1. (d) MCF-7 and T47D cells were transfected with Cyclin D1-luciferase and different FOXK2 constructs. Luciferase activity was measured either with or without pre-treatment of the cells with 10 nM E2 for 16 h. For comparison, the Cyclin D1-luc activity level of control cells was set to 1. (e) MCF-7 cells were transfected with His-Flag-FOXK2. The cells pre-treated with or without 10 nM E2 for 16 h and then subjected to real-time PCR to measure the mRNA levels of Cyclin D1 and GREB1. For comparison, Cyclin D1 and GREB1 mRNA levels of control cells were set to 1. All experiments were repeated at least three times. Each bar represents the mean ± SDs of three independent experiments. *, P < 0.05; **, P < 0.01. The full-length blot of Figure 5 is presented in Supplementary Figure 5.

FOXK2-promoted degradation of ERα was expected to have a negative effect on the transcriptional activity of ERα. Therefore, the effect of FOXK2 on the transcriptional activity of ERα was determined by using a reporter gene construct consisting of estrogen responsive element-luciferase (ERE-luc). MCF-7 and T47D cells were transfected with the ERE-luc construct and ERα only, or ERE-luc, ERα and FOXK2, and the level of reporter activity in these cells was measured following treatment with or without 17β-estradiol (E2). As shown in Fig. 5a, in the presence of E2 treatment, ERE-luc activity was highest when the cells overexpressed ERα alone. However, when these cells also overexpressed FOXK2, the level of ERE-luc activity was significantly reduced in a dose-dependent manner. Indeed, FOXK2 could both inhibit the transcriptional activity of ERα in the absence and presence of E2 treatment. These results corresponded to the reduction of ERα protein detected by Western blot (Fig. 3e). Cyclin D1 is a classical ERα-targeted gene and its promoter contains EREs. Similar results were obtained when the same experiment was carried out using MCF-7 and T47D cells that were transfected with Cyclin D1-luc, Flag-ERα and His-Flag-FOXK2 with or without E2 treatment (Fig. 5b). Next, we detected the effect of different FOXK2 constructs on the transcriptional activity of ERα using MCF-7 and T47D cells. Luciferase reporter assay showed that the transcriptional activity of ERα in cells transfected with FOXK2 FL significantly decreased compared to non-transfected cells; the transcriptional activity of ERα in cells transfected with FOXK2 Δ1 was similar to that in cells transfected with FOXK2 FL, whereas the transcriptional activity of ERα in cells transfected with FOXK2 Δ2 and Δ3 increased significantly, compared with that in cells transfected with FOXK2 Δ1 both in the cases of MCF-7 and T47D cells (Fig. 5c and 5d). Furthermore, we examined the ability of FOXK2 to regulate the expression of the well-established ERα-targeted genes (*Cyclin D1* and *GREB1*) in MCF-7 cells. Real-time PCR analysis showed that overexpression of FOXK2 reduced the mRNA levels of both *Cyclin D1* and *GREB1* (Fig. 5e). Taken together, these results showed that FOXK2 might suppress the transcriptional activity of ERα through promoting its degradation, and in doing so, it caused the down-regulation of the expression of ERα target genes.

### FOXK2 suppresses ERα-mediated growth of breast cancer cell

**Figure 6:**
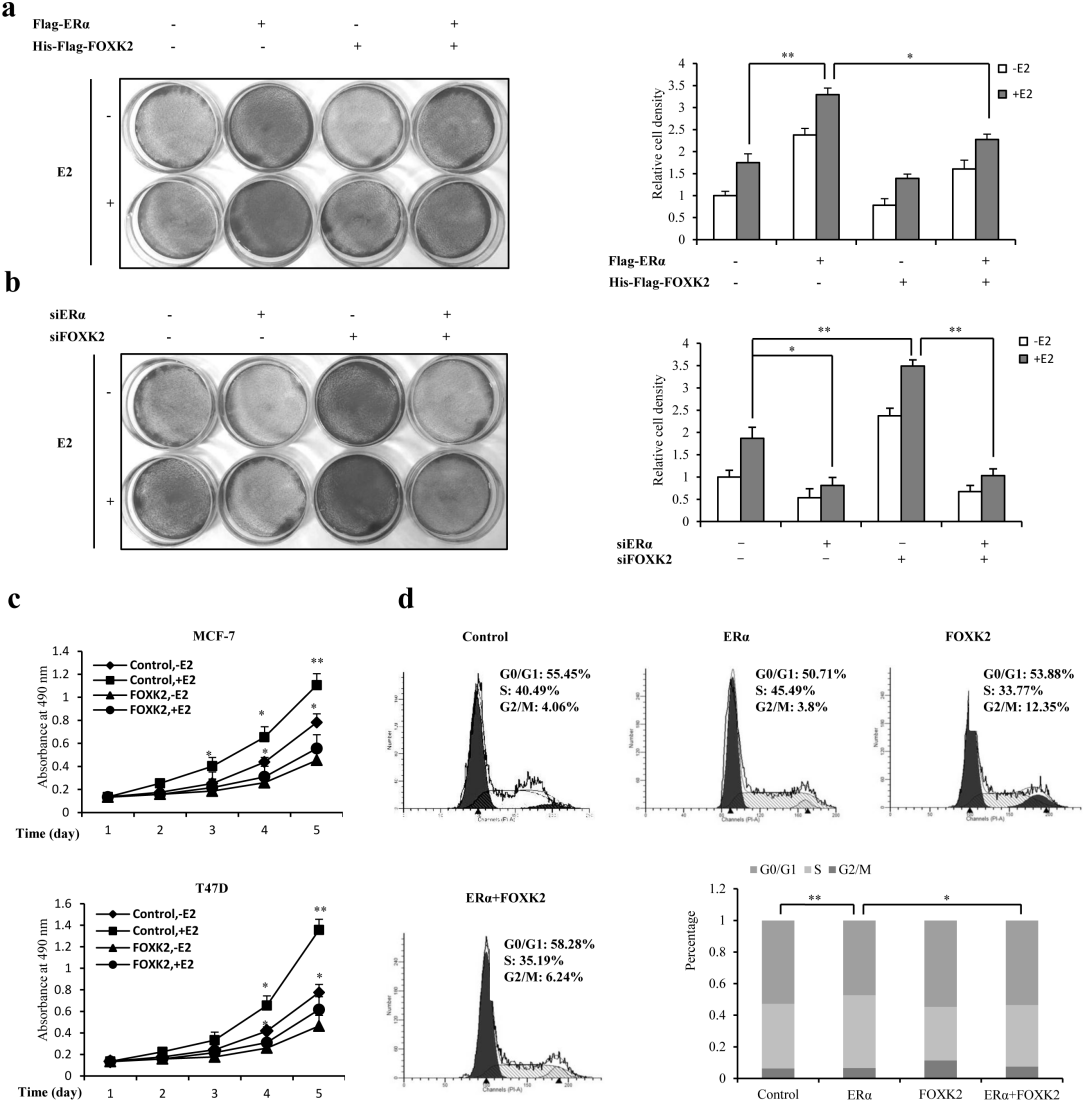
Effect of FOXK2 on ERα-mediated breast cancer cells proliferation. (a) MCF-7 Cells transfected with Flag-ERα, or with His-Flag-FOXK2, or with Flag-ERα and His-Flag-FOXK2 were stained with crystal violet after 8 days of growth (left panel). The right graph shows the relative cell density obtained from eight plates estimated by the software Imagepro-pus. For comparison, the number of control cells was set to 1. (b) MCF-7 Cells transfected with control siRNA (siControl), or with siERα, or with siFOXK2, or with siERα and siFOXK2 were stained with crystal violet after 8 days of growth (left panel). The right graph shows the relative cell density obtained from eight plates. The number of control cells was set to 1. (c) MCF-7 and T47D cells were transfected with His-Flag-FOXK2, and then treated without or with 10 nM E2 for the indicated times. The cells were then subjected to 3-(4,5-dimethylthiazol-2-yl)-2,5-diphenyltetrazolium bromide (MTT) assay performed according to the manufacturer's instructions (Key Gen). The absorption of control cells was set to 1. (d) MCF-7 cells were transfected with control vector, or with Flag-ERα, or with His-Flag-FOXK2, or with Flag-ERα and His-Flag-FOXK2. Flow cytometry analysis of cell-cycle distribution of MCF-7 cells after 36 h of growth in the presence of 10 nM E2. All experiments were repeated at least three times. Each bar represents the mean ± SDs of three plates *, P < 0.05; **, P < 0.01.

As FOXK2 was able to interact with ERα, and regulate its function, it may in fact affect ERα-mediated proliferation of breast cancer cells, especially since ERα is known to play a major role in the proliferation of breast cancer. Crystal violet staining assay showed that MCF-7 cells transfected with Flag-ERα produced more colonies than cells that were transfected with an empty vector (control cells) or cells that were transfected with both Flag-ERα and His-Flag-FOXK2 (Fig. 6a). In contrast, knockdown of ERα decreased, whereas knockdown of FOXK2 increased the colony numbers of MCF-7 cells compared with the control groups, whereas knockdown of FOXK2 increased the colony numbers of MCF-7 cells compared with control group. However when ERα was also knocked down, knockdown of FOXK2 had no effect on cell proliferation (Fig. 6b). We also examined the effect of FOXK2 on cell viability. Growth of both MCF-7 and T47D cells was inhibited when these cells were transfected with FOXK2 and cultured either in the absence of presence of E2 (Fig. 6c). The effect of FOXK2 on the cell-cycle was also investigated. MCF-7 cells transfected with either Flag-ERα or His-Flag-FOXK2 or both were subjected to flow cytometry analysis to evaluate the cell cycle profile of asynchronous cells. Cells transfected with ERα showed an overall increase in the percentage of cells in the S phase, with a corresponding reduction in the percentage of cells in G0/G1 phase compared with control cells (Fig. 6d). In contrast, the percentage of cells in the S phase decreased for cells transfected with FOXK2 decreased the percentage of S phase cells compared with control cells. When the cells were transfected with both ERα and FOXK2, the percentage of S phase cells decreased compared with cells only transfected with ERα. Taken together, these results suggested that FOXK2 could suppress the growth of breast cancer cells through its modulation of ERα.

## Discussion

Growing evidence has shown that ERα plays a key role in the initiation and development of breast cancer, and this has made ERα a valuable predictive and prognostic biomarker for the treatment of breast cancer^25^,^26^,^27^. However, much of the detailed mechanism involved in the regulation of ERα function is still unclear, and this appears to restrict our understanding of the pathogenesis of ERα-positive breast cancer. Thus it is important to gain further insight into how ERα function is regulated. In this study, we focused on the role of FOXK2 in ERα-positive breast cancer cells as this would allow us to investigate the connection between FOXK2 and ERα in breast cancer and to interpret this connection in terms of its significance in biological function.

The forkhead transcription factors are an evolutionarily conserved family of proteins. In mammals, there are over 40 different forkhead transcription factors, and these proteins control several cellular processes, including growth, development, proliferation and cell cycle through regulating the expression of their target genes^28^,^29^,^30^. Forkhead transcription factors also interact with other transcription factors, and regulate their functions, such as the co-association of FOXA1 with ER and AR^22^,^31^, FOXO3a with ERα and ERβ^7^, FOXM1 with Sp1^32^ and p53^33^, and FOXO1A with HoxA-11^34^. The data from breast tumor specimens that we analyzed indicated a negative correlation between FOXK2 and ERα (Fig. 1). Furthermore, the data from coimmunoprecipitation, mammalian two hybrid system and GST pull-down assay clearly revealed that FOXK2 interacted with ERα (Figs. 2a–e), although we could not conclude from our data whether FOXK2 and ERα directly interact with each other or via some an accessory element. The interaction was obvious and real, and subsequent reporter gene assay showed that FOXK2 suppressed the transcriptional activity of ERα and it achieved this through affecting its protein stability rather than its gene expression (Figs. 5a–d). The mechanism may stem from FOXK2 playing a structural role, such as stabilizing the protein complex, thereby making ERα more readily for ubiquitination. In the case of FOXO3a, its interaction with ERα has been demonstrated to occur via its FOX domain, and this interaction also results in the inhibition of ERα transcriptional activity^7^. However, whether FOXO3a affects ERα at the level of protein or gene was not demonstrated. We not only identified the exact domain of FOXK2 that interacted with ERα, but also showed that such interaction led to enhanced the degradation of ERα via the proteasome, and hence, its loss of transcriptional activity.

Ubiquitin-dependent protein degradation plays an important role in many basic cellular functions through regulating different cell regulators, such as tumor regulators, transcriptional factors and cell surface receptors^35^,^36^. Before the target protein is degradated by 26S proteasome, it must be attached conjugated to ubiquitin, a process that is catalyzed by an E3 ubiquitin ligase^37^,^38^,^39^,^40^. FOXK2 lacks the catalytic domains of ubiquitin E3 ligase, and does not have the function of ubiquitin E3 ligase. Thus we speculated that FOXK2 may increase the ubiquitination of ERα through regulating the interaction between ERα and its E3 ligases. Indeed, FOXK2 interacted with BARD1 and thus, the BRCA1/BARD1 complex could be responsible for the degradation of ERα. If so, then FOXK2 would appear to mediate the degradation of ERα via an accessory protein, BARD1. Furthermore, FOXK2 interacted with ERα and BARD1 at different domains (Figs. 2e and 4d), suggesting that the interaction was rather specific in each case. The involvement of BARD1 in FOXK2-regulated ERα activity was clearly supported by the data which showed that overexpression of FOXK2 promoted the interaction between BARD1 and ERα (Fig. 4h), whereas knockdown of FOXK2 weakened their interaction (Fig. 4i).

ERα is a member of the steroid hormone receptor superfamily of ligand-activated transcription factors. As a transcription factor, ERα plays a crucial role in regulating the normal function of reproductive tissues and proliferation of epithelial cells. It also plays an important role in the genesis and malignant progression of breast cancer. Aberrant activation of ERα contributes to tumorigenesis of the breast by up-regulating its target genes such as *TFF1*, *SDF-1*, *Cyclin D1* and *GREB1*^41^,^42^,^43^,^44^,^45^. Among them, *Cyclin D1* is a major regulator that governs the entrance of a cell into the proliferative stage of the cell cycle, and its expression is regulated by ERα, which mediates its proliferative action on mammary cancer cells^44^. Thus, there is a strong correlation between increased proliferative response and increased levels of *Cyclin D1* mRNA, and this could be seen from the increased levels of ERα in MCF-7 cells that stably expressed ERα compared to control cells (no overexpression of ERα^46^. Our data here that the mRNA level of *Cyclin D1* was up-regulated in MCF-7 cells following treatment with E2, whereas this up-regulation was inhibited when the cells overexpressed FOXK2. The up-regulation of *GREB1* mRNA level was also inhibited by FOXK2 (Fig. 5e), and was consistent with the result obtained for *Cycline D1*. This indicated that FOXK2 could suppress the transcriptional activity of ERα, which would effectively down-regulate the expression of genes that are regulated by ERα.

Since FOXK2 could act as a negative regulator of ERα, we expected it to play a role in ERα-mediated cell proliferation. According to our data, either overexpression of ERα alone or knockdown of FOXK2 in MCF-7 cells could result in significant increases in cell number compared to control cells (no overexpression or knockdown of exogenous ERα or FOXK2) (Fig. 6a and 6b). This clearly showed that increase in the level of ERα activity resulting either from increased expression of the gene from exogenous source or from crippling FOXK2 (which had the effect of amplifying ERα activity) would ultimately lead to increased cell growth. A similar trend was observed in the cell viability assay (Fig. 6c). Furthermore, FOXK2 appeared to suppress ERα-mediated proliferation of breast cancer cells through inhibiting cell cycle progression (Fig. 6d).

In conclusion, we showed in this study that FOXK2 negatively regulated the function of ERα through enhancing its degradation via the proteasome, and identified the ubiquitin E3 ligase BRCA1/BARD1 complex as an important contributing factor. This negative regulation of ERα by FOXK2 would disrupt the ERα-mediated cell growth, and in the case of breast cancer cells, it would mean a reduction in cell proliferation and possibly, the spread of cancer cells. However, since ERα is also needed for the normal functioning of the cell, targeting it with a negative regulator gene that would result in its degradation is not an ideal strategy for combating breast cancer, even for ERα-positive breast cancer. Therefore, further work is desirable, such as more in depth investigation of the molecular interaction between FOXK2 and ERα and their effect on normal cells.

## Methods

### Ethics statement

The study involving human participants was approved by the institutional review board of Dalian University of Technology. Written consent was obtained from all the participants. The methods were carried out in accordance with the approved guidelines. All clinical research was performed on the basis of the principles expressed in the Declaration of Helsinki.

### Plasmids and antibodies

His-Flag-tagged FOXK2 and EGFP-FOXK2 were gifts kindly provided by Dr. Andrew D. Sharrocks (University of Manchester). HA-BARD1 was a gift kindly provided by Tomohiko Ohta (St. Marianna University). Cyclin D1-luc was kindly provided by Dr. Robert Weinberg (Whitehead Institute for Biomedical Research). Flag-tagged full-length and truncated FOXK2 (Δ1, Δ2, Δ3 and Δ4) were constructed according to standard PCR-based cloning procedures using His-Flag-FOXK2 as templates. PCR fragments were inserted into pcDNA3.1-3×Flag at the *Bam*HI and *Hind*III sites. Plasmid encoding GST-fusion protein was prepared by standard PCR methods using His-Flag-FOXK2 as templates, and the PCR fragment was cloned in frame into pGEX-4T3 (Amersham Pharmacia) at the *Bam*HI and *Sal*I sites. SMARTpool® siRNAs (Control, ERα and FOXK2) with four individual siRNAs targeting a single gene were obtained from Thermo (USA).

Rabbit polyclonal anti-Flag, anti-HA, anti-ERα, anti-GFP, anti-IgG and mouse monoclonal anti-Actin antibodies were obtained from Santa Cruz Biotechnology (Santa Cruz, CA). Mouse monoclonal anti-ERα and anti-GST antibodies were obtained from Millipore. Mouse monoclonal anti-HA and anti-GFP antibodies were obtained from GeneTex. Rabbit polyclonal anti-c-Myc and mouse monoclonal anti-Flag (M2) antibodies were purchased from Sigma. Goat polyclonal anti-FOXK2 (ILF1, ab5298) and rabbit polyclonal anti-FOXK2 (ILF1, ab84761) antibodies were obtained from Abcam. Rabbit polyclonal anti-BARD1 antibody was obtained from BIOSS (Beijing, China). Rabbit polyclonal anti-MDM2 antibodies were obtained from Sangon (Shanghai, China). Cycloheximide was obtained from Sigma, and MG132 was obtained from Merck.

### Cell culture and transfection

HEK 293T, MCF-7, Bcap-37, MDA-MB-231 and T47D cells had been used in our previous study^47^,^48^. ZR-51-30 and BT474 cells were obtained from the cell bank of the Shanghai branch of Chinese Academy of Sciences. Unless other stated, all cell cultures were incubated at 37°C in the presence of 5% CO_2_. HEK 293T, MCF-7, Bcap-37, MDA-MB-231 cells were cultured in Dulbecco's modified Eagle's medium (DMEM, Invitrogen) containing 10% fetal bovine serum (Hyclone) and penicillin-streptomycin (100 U/ml penicillin and 0.1 mg/ml streptomycin). ZR-51-30 and BT474 cells were maintained in Roswell Park Memorial Institute (RPMI) 1640 medium supplemented with 10% fetal bovine serum, penicillin-streptomycin. T47D cells were maintained in Roswell Park Memorial Institute (RPMI) 1640 medium supplemented with 10% fetal bovine serum, penicillin-streptomycin and insulin (5 g/ml). For E2 stimulation experiments, MCF-7 and T47D cells were subjected to serum starvation for 24 h in 2% charcoal-stripped fetal bovine serum (Gibico) and phenol red free medium, and then treated with or without 10 nM E2 for 16 h. Lipofectamine 2000 (Invitrogen) was used for cell transfection. Corresponding empty vectors were used in each transfection experiment to guarantee the same amount of plasmids for all parallel groups. All transfection experiments were transient transfection.

### Immunoprecipitation and Western blot

Cells were harvested and then lysed in a cold hypotonic buffer containing 50 mM Tris-HCl (pH 8.0), 150 mM NaCl, 0.1% SDS, 1% NP-40, 0.5% sodium deoxycholate and a mixture of protease inhibitors. After centrifugation at 10000 × g/4°C for 10 min, the supernatant was incubated with the desired antibody or with control IgG and protein A-Sepharose (Amersham Biosciences) or protein G-Sepharose (Santa Cruz, CA) at 4°C for overnight. After that, the sample was centrifuged at 5000 × g/4°C for 10 min and the pellet was washed twice with Washing Buffer I (50 mM Tris–HCl [pH 7.5], 150 mM sodium chloride, 1% NP-40 and 0.05% sodium deoxycholate) and once with Washing Buffer II (50 mM Tris–HCl [pH 7.5], 500 mM sodium chloride, 0.1% NP-40, and 0.05% sodium deoxycholate), and then subjected to SDS-PAGE. After electrophoresis, protein bands in the gel were transferred to PVDF membrane (Millipore), and probed with the specified primary antibody, followed by the appropriate secondary antibody, and then visualized using the enhanced chemiluminescence detection reagents (Thermo). Immunoblot data were quantified by scanning the appropriate bands of interest and plotted as relative density of gray scale. Re-immunoprecipitation was conducted as previously described^49^.

### Immunofluorescence staining

MCF-7 cells were cultured for overnight on cover slips. After 24 h, the cells were fixed in 1% paraformaldehyde for 15 min at room temperature, permeabilized with methanol for 40 min at −20°C and then blocked with 0.8% BSA for 1 h at 4°C. The cells were then incubated with appropriate antibodies at 4°C for overnight, followed by further incubation with TRITC-conjugated anti-rabbit IgG for 1 h. The cover slips were then mounted on glass slides with mounting medium containing 49, 6-diamidino-2-phenylindole (DAPI). Images were taken with a confocal fluorescence microscope (Olympus FV1000-IX81, Tokyo, Japan).

### Immunohistochemical assay

A total of 53 breast cancer specimens were obtained from female patients of Han Chinese descent, with a median age of 59.1 years, ranging from 39 to 78 years. Out of 53 specimens, 27 were ERα-positive, and 26 were ERα-negative as determined by clinical diagnosis performed by Qiqihar Medical University. Sections (4 micrometers thickness) of the obtained specimens were cut out and used for immunohistochemical analysis. The immunohistochemical assay kit was obtained from Maixin Bio. Immunohistochemical assay was conducted as previously described^50^. The primary antibodies used in immunohistochemical assay were rabbit anti-human FOXK2 and mouse anti-human ERα. The levels of FOXK2 and ERα expression were quantified according to their H-scores^51^. ERα was considered positive if the H-score was more than 1^52^,^53^. The median H-score of all samples was used as a cutoff for grouping the samples into high or low FOXK2 expression category^54^.

### Luciferase reporter assay

Cells were cultured in a 24-well plate for 24 h. The cells were then transfected with the appropriate plasmid construct using Lipofectamine 2000 (Invitrogen). Eighteen hours after transfection, the medium was replaced with phenol red-free medium containing 2% charcoal-stripped fetal bovine serum for 24 h, followed by treatment with or without 10 nM E2 for 16 h. The cells were harvested and Luc reporter assay was performed in accordance to the manufacturer's instructions (Promega, Madison, WI, USA).

### Mammalian two hybrid assay

The checkmate TM mammalian two-hybrid system was obtained from Promega. ERα and FOXK2 were subcloned into *Bam*HI–*Sal*I cut pACT and pBIND, respectively.

### GST pull-down assay

The GST alone and GST fusion protein were expressed in *E. coli* BL21 (Takara), and purified by Pierce GST Spin Purification Kit (Thermo scientific). GST pull-down assay was performed using a Pierce GST Protein Interaction Pull-Down Kit (Thermo scientific). The purified GST-tagged fusion protein (BAIT) was immobilized on the Pierce Spin Column. MCF-7 cells were lysed in pull-down lysis buffer containing DNase (Takara). The supernatant was loaded onto the Pierce Spin Column, and then incubated at 4°C for 2 hour with gentle agitation. The column was centrifuged at 1250 × g for 1 minute and the flow through was discarded. Then the column was washed five times using wash solution. Elution buffer was added to the column followed by 5-min incubation with gentle agitation. After that, the column was centrifuged at 1250 × *g* for 1 minute, and the eluent was subjected to Western blot assay.

### Cell growth assays

MTT and Flow Cytometry assays were performed as previously described^47^,^54^. For Flow Cytometry assay, MCF-7 cells transfected with different plasmids were stained with propidium iodide (PI) (BD Pharmingen, CA). Experimental data were collected by FACSCalibur (BD Biosciences, San Jose, CA, USA). Cell cycle profiles were determined using ModFit LT (BD Biosciences). For crystal violet staining assay, MCF-7 cells transfected with the appropriate plasmids were transferred to 35 mm plate, and were cultured until the recognizable clones appeared. Then, the cells were stained with crystal violet for 30 min at room temperature.

### Real-time PCR

MCF-7 cells were transfected with appropriate plasmids. Twenty four hours after transfection, the cells were replaced with phenol red-free medium containing 2% charcoal-stripped fetal bovine serum for 24 h, then treated with or without 10 nM E2 for 16 h. Total RNA was isolated from the cells using TRIzol reagent (Takara), and then subjected to reverse transcription with oligo(dT)15. The mRNA levels of ERα, Cyclin D1, GREB1 and GADPH (as an internal control) were quantitated by real-time PCR using Corbett Research RG 3000 analyzer (Australia), Maxima SYBR Green/ROX qPCR (Thermo Scientific). The following primer sequences were used: ERα: 5′-ACTCGCTACTGTGCAGTGTGCAAT (forward) and 5′-CCTCTTCGGTCTTTTCGTATCCCA (reverse); *Cyclin D1*: 5′-GCTGCTCCTGGTGAACAAGC (forward) and 5′-AAGTGTTCAATGAAATCGTGCG (reverse); *GREB1*: 5′-CAGGCTTTTGCACCGAATCT (forward) and 5′-CAAAGCGTGTCGTCTTCAGCT (reverse); *GADPH*: 5′-GGGTTGAACCATGAGAAGT (forward) and 5′-GACTGTGGTCATGAGTCCT (reverse). The mRNA levels of *ERα*, *Cyclin D1* and *GREB1* were normalized against *GAPDH*, which served as an endogenous control. Each gene was measured in triplicate.

### Statistical analysis

A Chi-square (χ^2^) test was used to examine the correlation between FOXK2 and ERα gene expression in breast cancer tissues from 53 patients. All statistical analyses of other data were performed with ANOVA, followed by the Bonferroni test for pairwise comparisons^55^,^56^. Data were given as means ± SDs, and significance was considered at the P value < 0.05 level.

## Author Contributions

Y.L., X.A. and H.W. conceived and designed the experiments. Y.L., X.A., Z.J., X.B., Z.X., G.H., X.J. and M.C. performed the experiments. Y.L., X.A. and H.W. analyzed the data. Y.L., X.A., Z.J., X.B., Z.X., G.H., X.J. and M.C. contributed reagents/materials/analysis tools. Y.L., X.A. and H.W. wrote the paper. All authors have read and approved the final manuscript.

## Supplementary Material

Supplementary Information Supplementary Information
