# Supplementary material for: FOXK2 Transcription Factor Suppresses ERα-positive Breast Cancer Cell Growth Through Down-Regulating the Stability of ERα via mechanism involving BRCA1/BARD1
Source: Sci Rep. 2015 Mar 5;5:8796. doi: 10.1038/srep08796 (PMC4350111; doi:10.1038/srep08796)
Supplement: Supplementary Information [file srep08796-s1.pdf]

Supplementary Information

FOXK2 Transcription Factor Suppresses ERα-positive Breast Cancer Cell Growth Through Down-Regulating the Stability of ERα via mechanism involving BRCA1/BARD1.

Ying Liu †, Xiang Ao †, Zhaojun Jia, Xiaoyan Bai, Zhaowei Xu, Gaolei Hu, Xiao Jiang, Min Chen and Huijian Wu*

School of Life Science and Biotechnology, Dalian University of Technology, Linggong road 2, Dalian 116024, Liaoning, People's Republic of China.

† These authors contributed equally to this study.

***Corresponding authors: Huijian Wu, PhD**

Supplementary information includes six Figures.

Supplementary Figure 1

Figure 1c

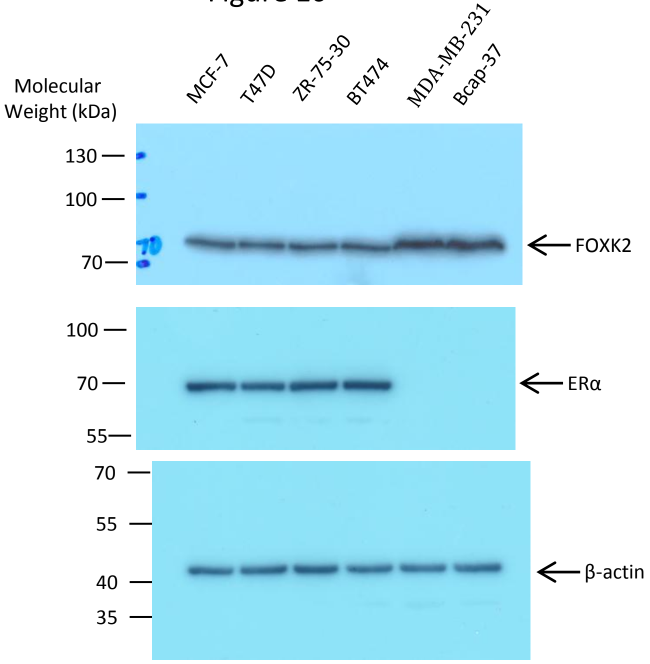

Figure 1. The full-length blot of Figure 1 including molecular size maker. Numbers to the left indicate the positions of size markers (in kDa) and the arrows indicate the band of interest.

Supplementary Figure 2

Figure 2a

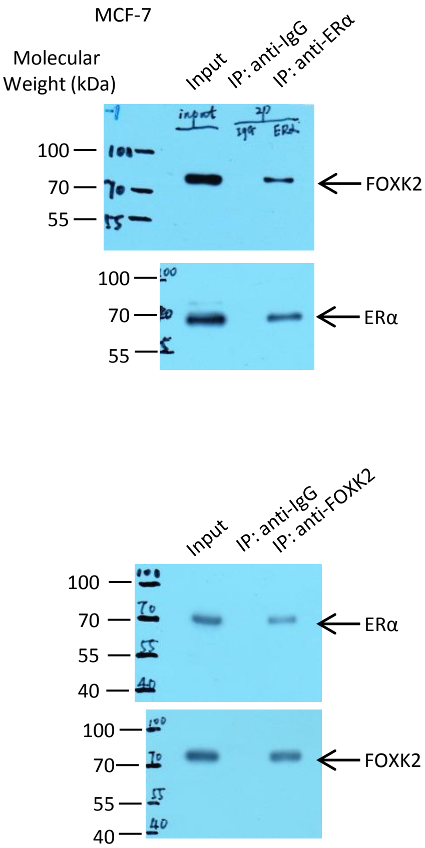

Figure 2b

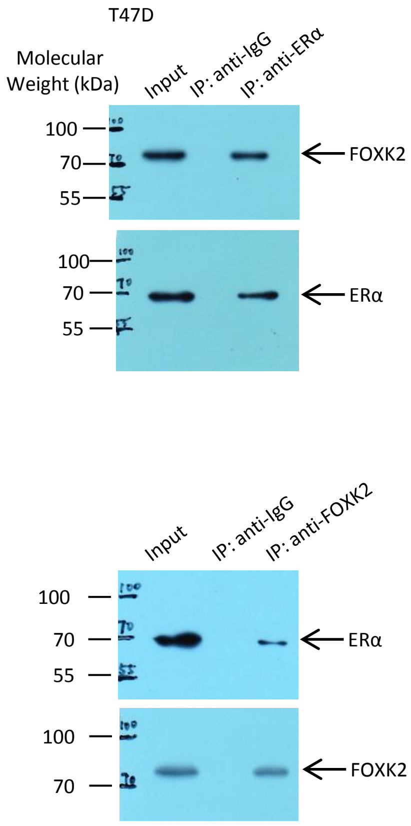

Figure 2c

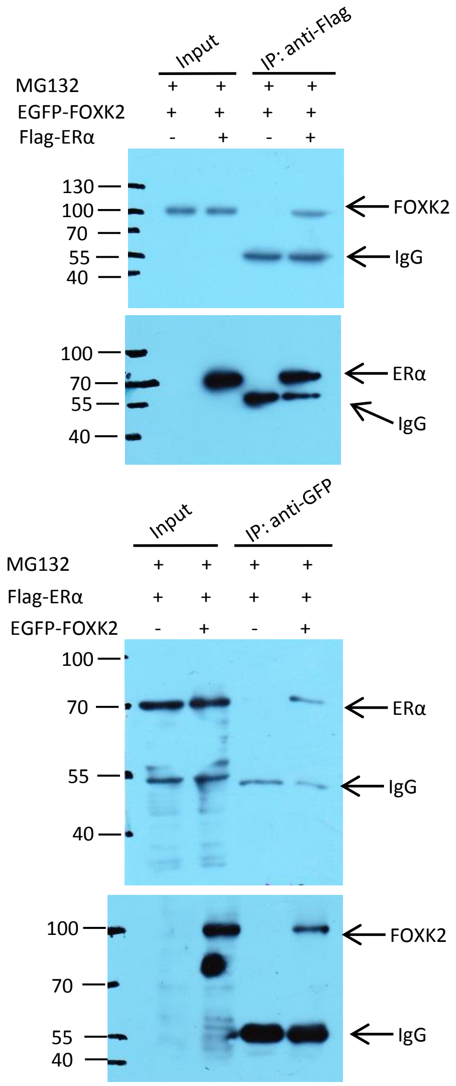

Figure 2e

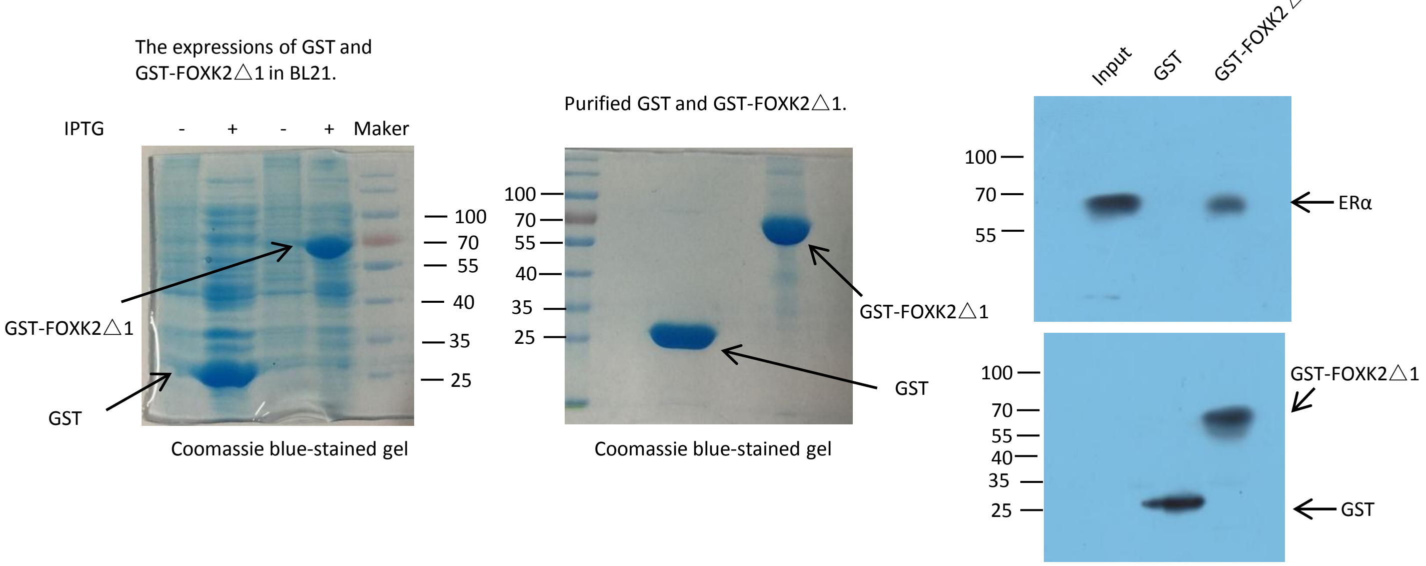

Figure 2f

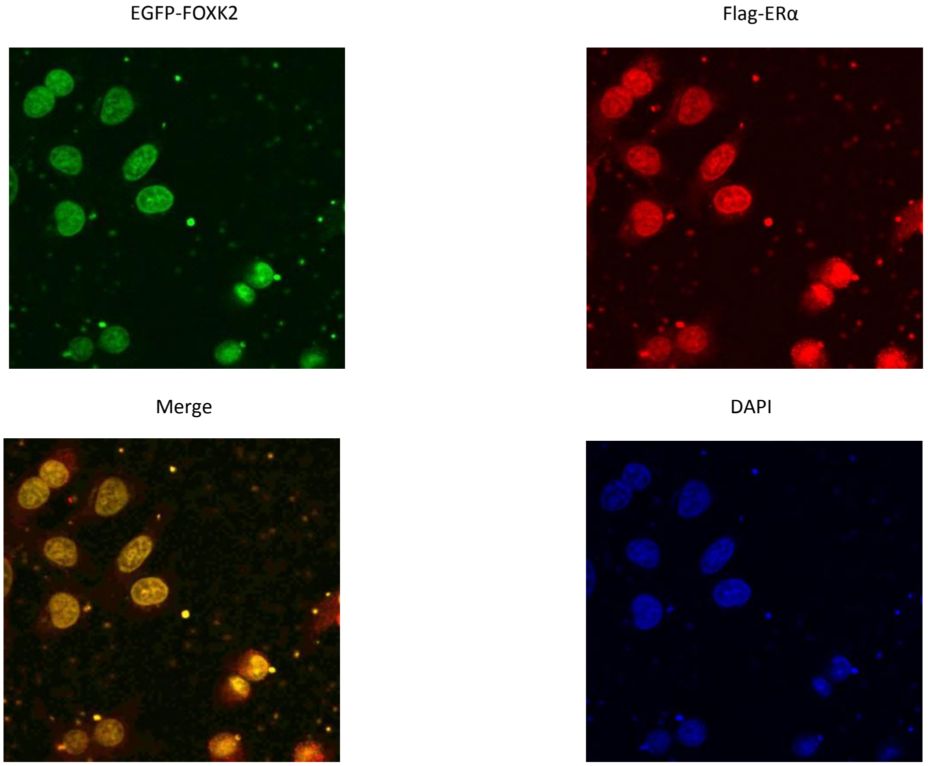

Figure 2g

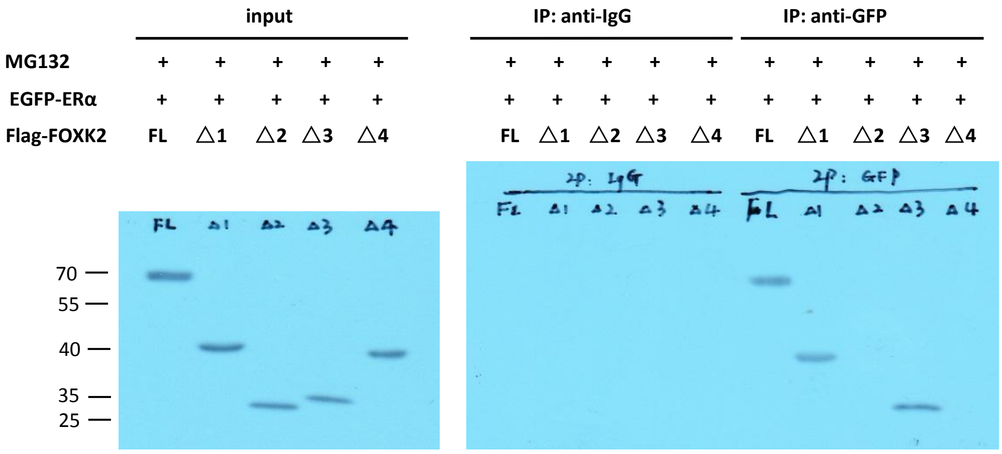

Figure 2. (a-d) The full-length blot of Figure 2 (a-d) including molecular size maker. Numbers to the left indicate the positions of size markers (in kDa) and the arrows indicate the band of interest. (e) Left panel shows the expression of GST alone and GST fusion FOXK2 △1 induced by IPTG. Middle panel shows the expression of GST alone and GST fusion FOXK2 △1 purified by Pierce GST Spin Purification Kit. Right panel shows the interaction of FOXK2△1 with ERα *detected* by Western blot. (f) The unprocessed image of Figure 2F.

Supplementary Figure 3

Figure 3a

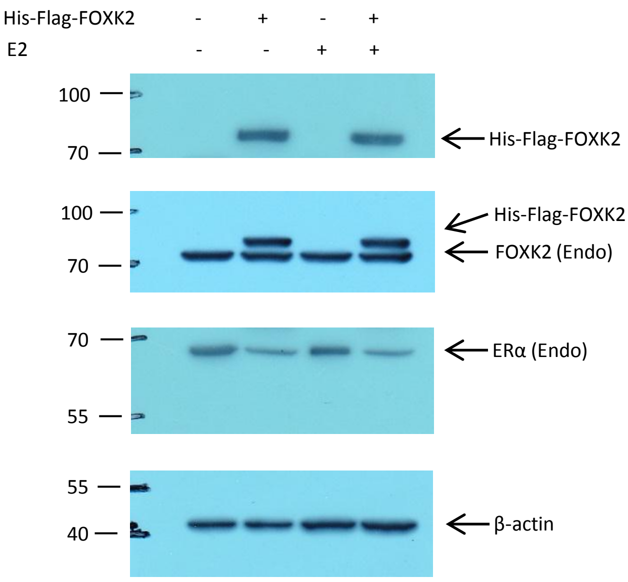

Figure 3c

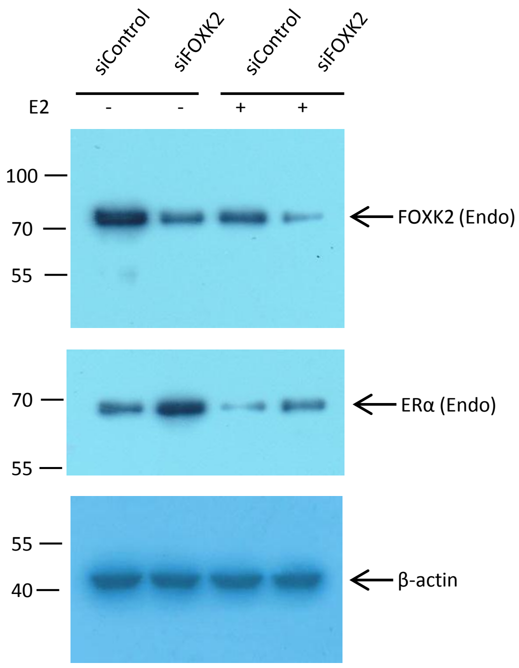

Figure 3e

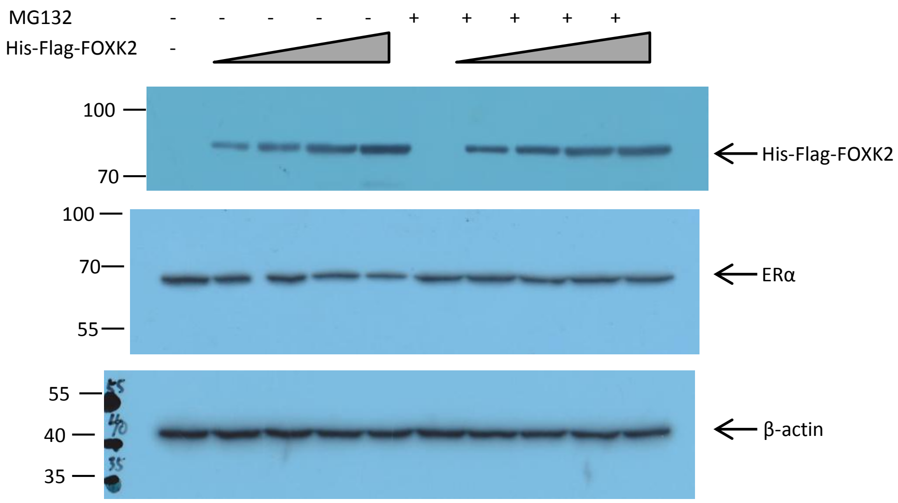

Figure 3f

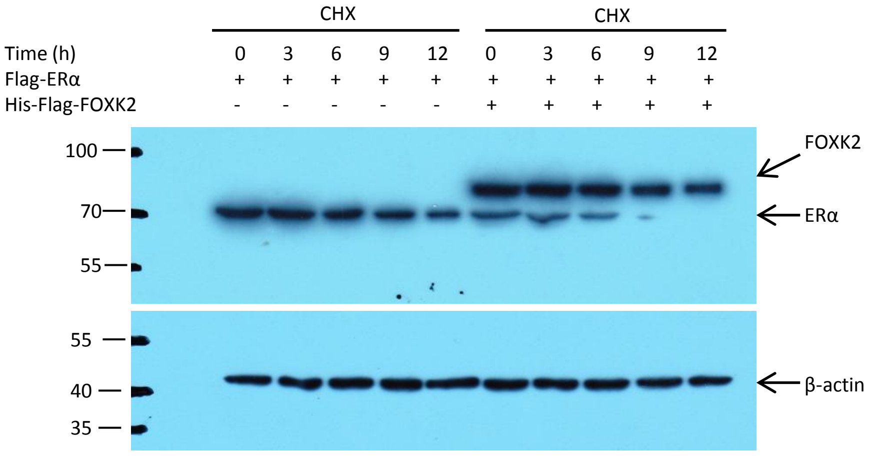

Figure 3g

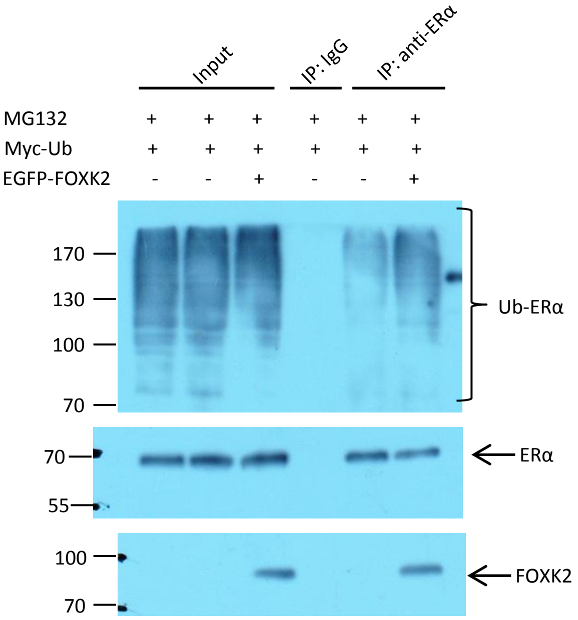

Figure 3. The full-length blot of Figure 3 including molecular size maker. Numbers to the left indicate the positions of size markers (in kDa) and the arrows indicate the band of interest.

Supplementary Figure 4

Figure 4a

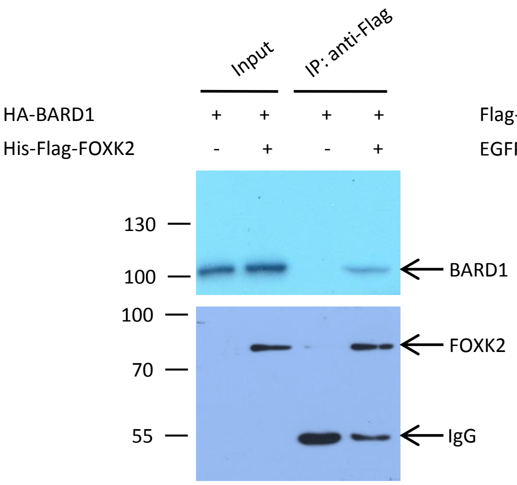

Figure 4b

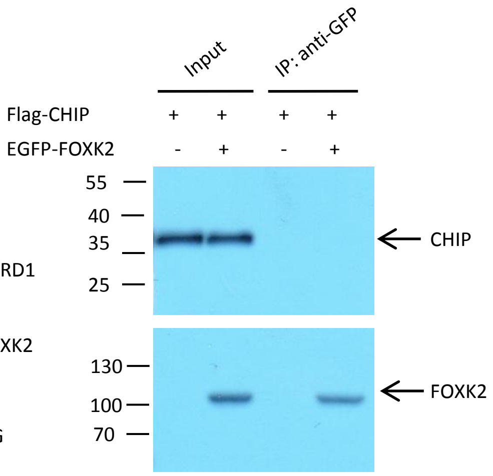

Figure 4c

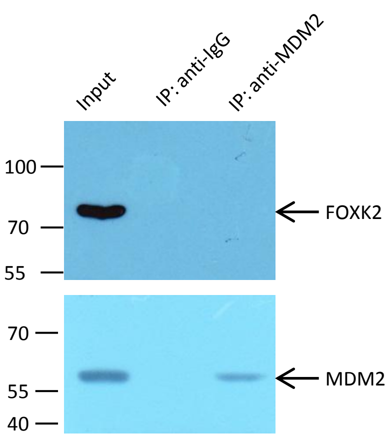

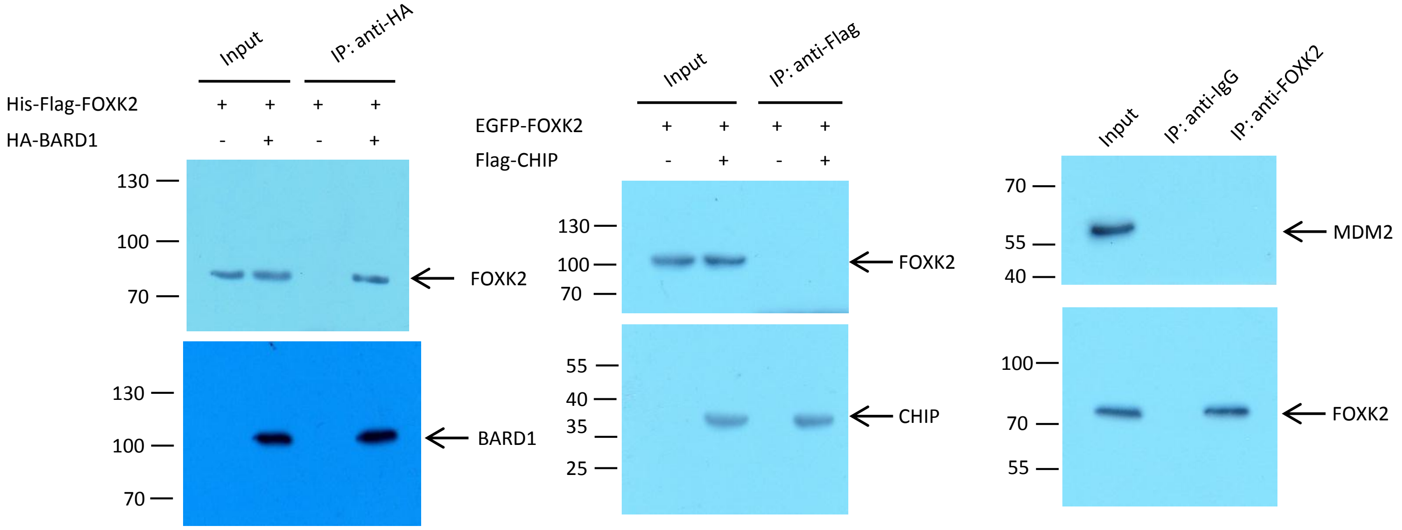

Figure 4d

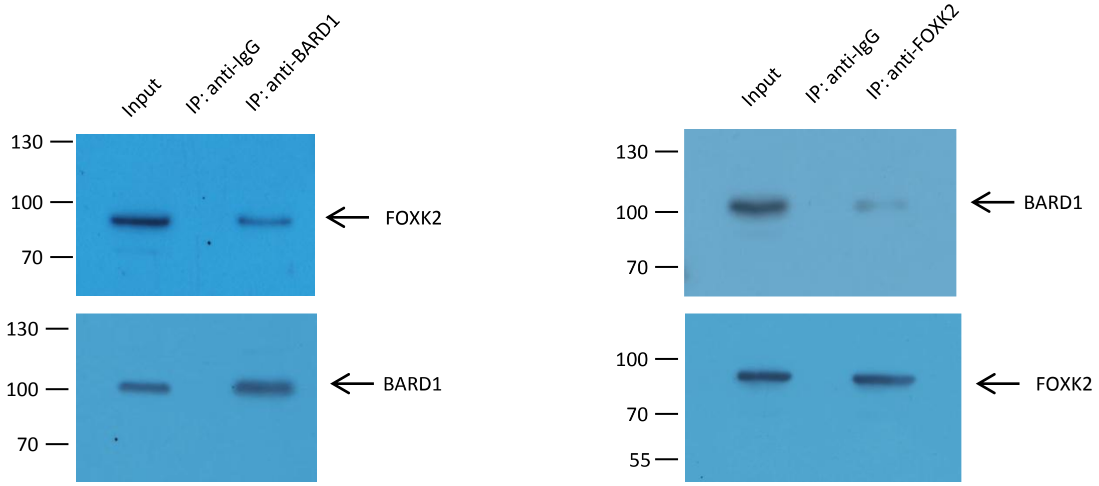

Figure 4e

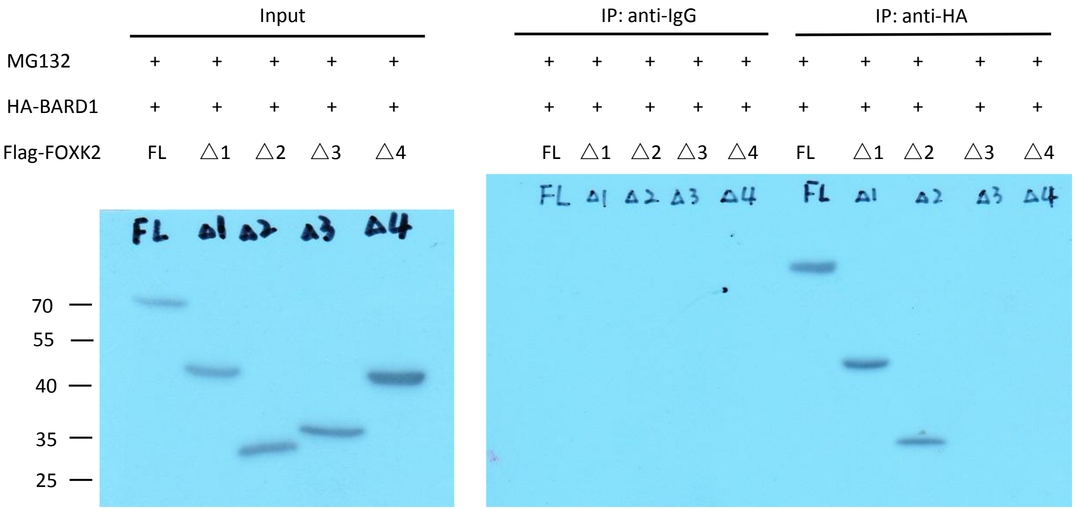

Figure 4f

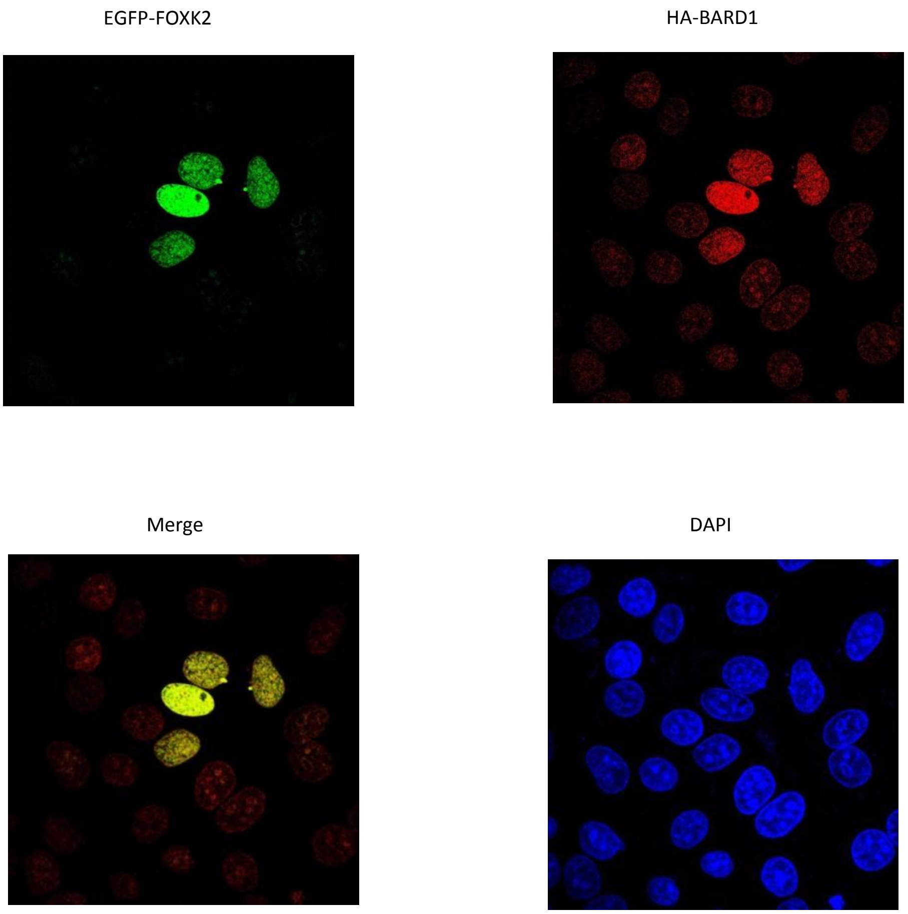

Figure 4g

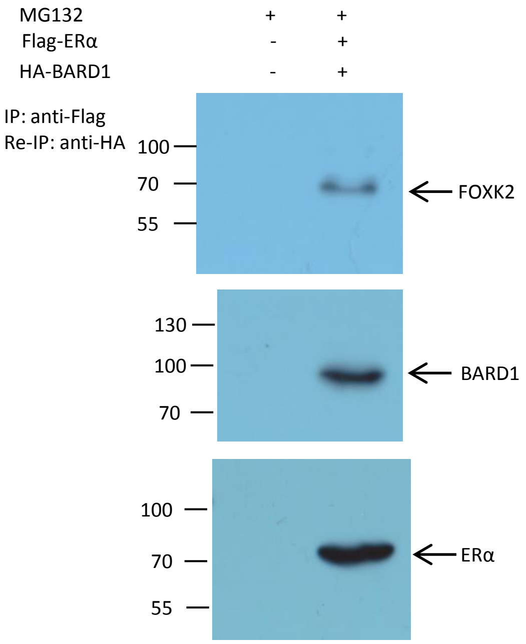

Figure 4h

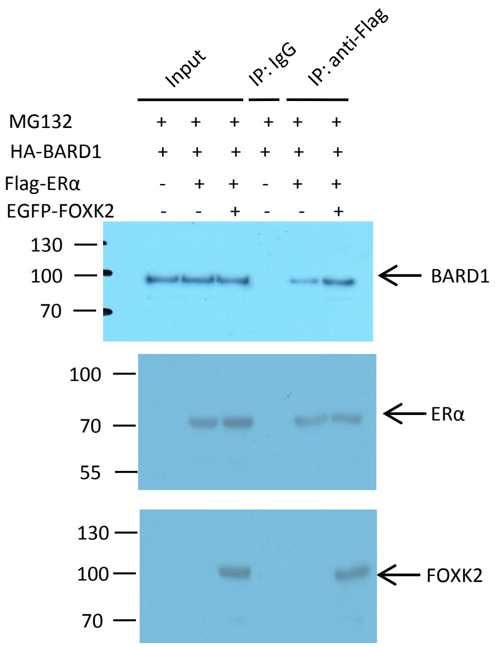

Figure 4i

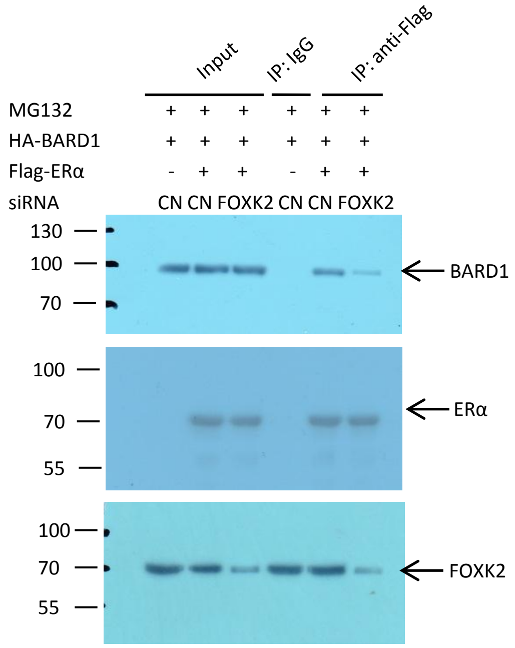

Figure 4j

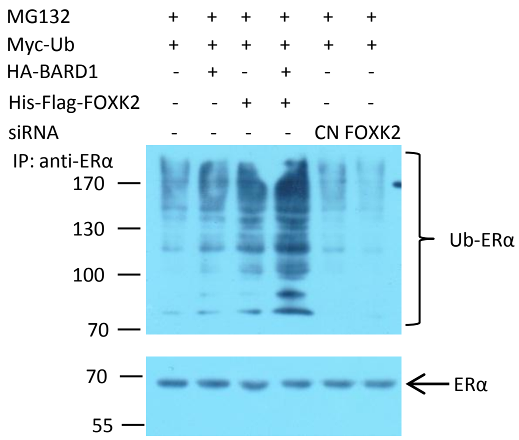

Figure 4. (a-d) The full-length blot of Figure 4 (a-d) including molecular size maker. Numbers to the left indicate the positions of size markers (in kDa) and the arrows indicate the band of interest. (e) The unprocessed image of Figure 2e. (f-h) The full-length blot of Figure 4 (f-h)

Supplementary Figure 5

Figure 5a

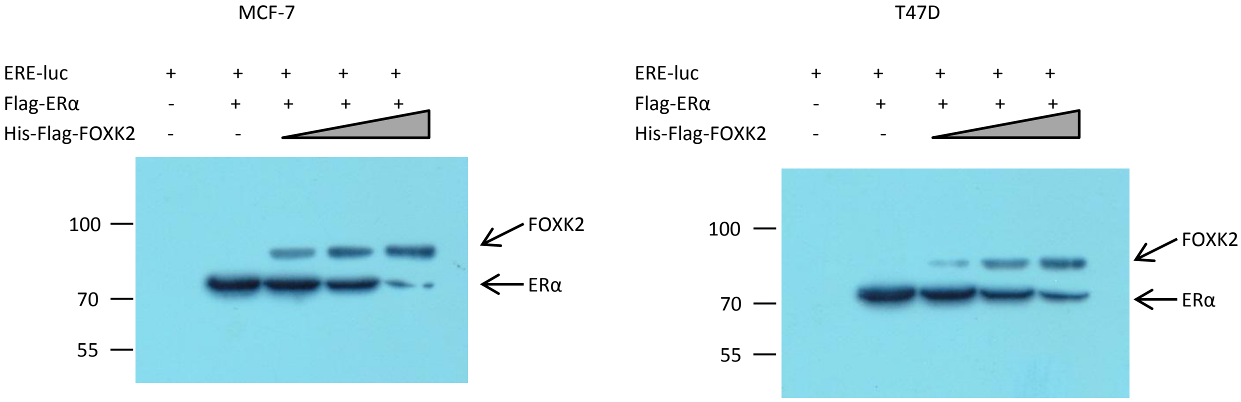

Figure 5b

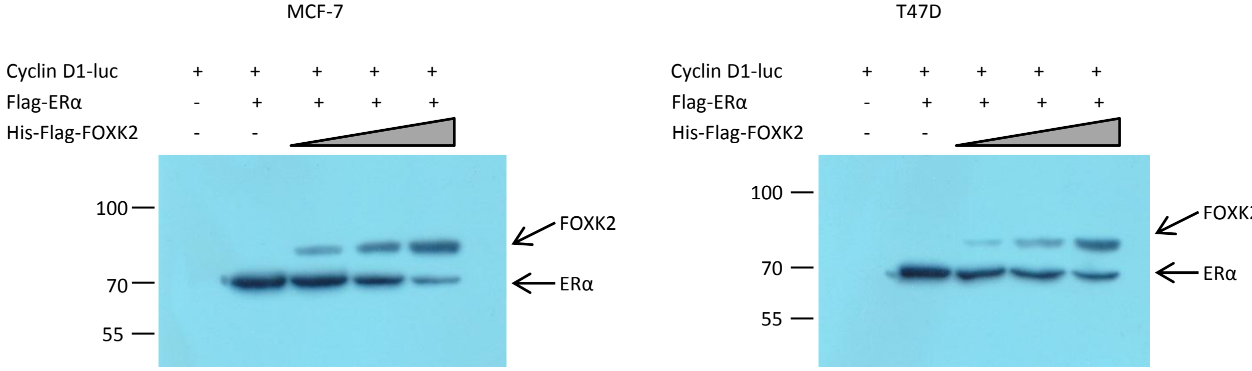

Figure 5. The full-length blot of Figure 5 including molecular size maker. Numbers to the left indicate the positions of size markers (in kDa) and the arrows indicate the band of interest.

Supplementary Figure 6

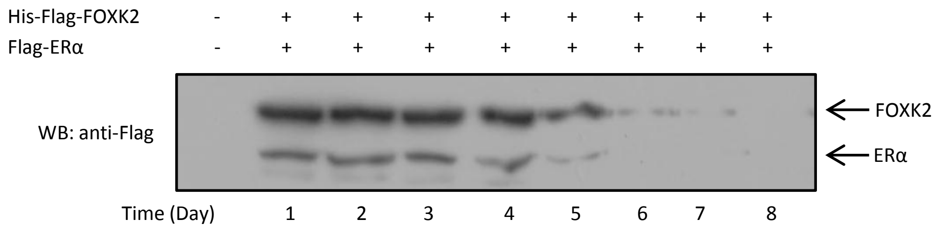

Figure 6. The expression of Flag tagged proteins in MCF-7 cells at different time points. MCF-7 cells transfected with His-Flag-FOXK2 and Flag-ERα were harvested at different time points, and were subjected to Western blot analysis with anti-Flag antibody.
